# Cellular fractionation reveals transcriptome responses of human fibroblasts to UV-C irradiation

**DOI:** 10.1038/s41419-022-04634-x

**Published:** 2022-02-24

**Authors:** Jiena Liu, Zhenzhen Wu, Jin He, Yuming Wang

**Affiliations:** 1 Institute of Neuroscience and the Second Affiliated Hospital of Guangzhou Medical University, Key Laboratory of Neurogenetics and Channelopathies of Guangdong Province and the Ministry of Education of China, Guangzhou, 510260 China; 2 School of Basic Medical Sciences, Guangzhou Medical University, Guangzhou, 511436 China; 3 School of Management, Guangdong University of Technology, Guangzhou, 510520 China

**Keywords:** Nucleotide excision repair, Long non-coding RNAs

## Abstract

While cells activate a multifaceted DNA damage response to remove transcription-blocking DNA lesions, mechanisms to regulate genome-wide reduction of RNA synthesis and the paradoxical continuous loading of RNAP II at initiation sites are still poorly understood. Uncovering how dramatic changes to the transcriptional program contribute to TC-NER (transcription-coupled nucleotide excision repair) is important in DNA repair research. However, the functional significance of transcriptome dynamics and the mechanisms of chromatin attachment for thousands of unstudied human lncRNAs remain unclear. To address these questions, we examined UV-induced gene expression regulation in human fibroblasts by performing RNA-seq with fractionated chromatin-associated and cytoplasmic transcripts. This approach allowed us to separate the synthesis of nascent transcripts from the accumulation of mature RNAs. In addition to documenting the subcellular locations of coding transcripts, our results also provide a high-resolution view of the transcription activities of noncoding RNAs in response to cellular stress. At the same time, the data showed that vast majority of genes exhibit large changes in chromatin-associated nascent transcripts without corresponding changes in cytoplasmic mRNA levels. Distinct from protein-coding genes that transcripts with shorter length prefer to be recovered first, repression of lncRNA transcription after UV exposure is inactivated first on noncoding transcripts with longer length. This work provides an updated framework for cellular RNA organization in response to stress and may provide useful information in understanding how cells respond to transcription-blocking DNA damage.

## Introduction

The response to DNA damage intersects with many other physiological processes in the cell, including initiation of DNA repair, chromatin remodeling, regulation of transcription and translation, and the cell cycle to contend with the challenge. Accumulation of nuclear DNA damage caused by DNA repair deficiency has been associated with accelerated aging disorders and normal aging [1–3]. Certain damaging lesions, such as UV-induced pyrimidine dimers, strongly block RNA polymerases, necessitating the coordination of the transcription-coupled nucleotide excision repair (TC-NER) with the remodeling of the elongating transcriptional machinery [4, 5]. Accordingly, transcription shutdown is not simply a consequence of the physical arrest of RNA polymerase II progression by DNA lesions, but also could be elicited by signaling in trans, as several lines of evidence have clearly showed disturbed transcription activity on undamaged DNA templates in UV-irradiated cells [6–8]. While to date many of the specific molecular events are not fully elucidated.

Two of the factors CSA and CSB involved in the TC-NER pathway were initially characterized by their association with an inherited syndrome, namely Cockayne syndrome (CS) [9]. Several current studies provide molecular insights into the concerted action of CSA and CSB in regulating the timing of transcription arrest and restore. The model proposes that CSB recruits CSA to TSS sites, mediating the ubiquitination and degradation of the ATF3 repressor on chromatin to elicit the restart of RNA synthesis after genotoxic stress [10]. Meanwhile, CSB recruits the PAF1 complex onto RNAP II paused at TSS sites to promote pause release and stimulate productive elongation throughout genes [11]. Although the precise mechanisms are about to be disclosed with an increasing number of DNA damage-induced factors involved in the processive transcription to be identified, insight into the features of RNA products from transcription restore after genotoxic stress is limited. Recent studies have revealed that continuous engagement of RNAP II molecules ensures maximal transcription-driven repair throughout expressed genes [12]. In this respect it is interesting to evaluate the impact of these active TSS sites after UV on the genome-wide processive transcription elongation after DNA repair. In support of this concept, a number of recent papers have shown that RNA pathways (including RNA synthesis and processing) in turn affect the cellular response to DNA damage [13]. Although it has been suggested that modulation of specific transcriptional programs by lncRNAs might be another mechanism that regulates the DNA damage responses, the lncRNA transcriptional portrait in response to genotoxic stress is largely unknown.

Here we sought to capture the transcription shutdown and restart and isolate the set of lncRNAs that are likely to function at the chromatin interface by using biochemical fractionation of the cellular compartments coupled to RNA-seq. Unexpectedly, vast majority of protein-coding genes exhibit large changes in chromatin-associated nascent transcripts without corresponding changes in cytoplasmic mRNA levels. We find that the bulk of mRNAs recovered from the stalled RNAP II are relatively short, suggesting a pervasive mechanism of UV-induced specific transcription elongation. By contrast, transcription of noncoding RNAs with longer length is preferred to be restored first in response to cellular stress. Yet, as a master regulator of gene expression, CSB protein does not display robust control on transcription levels of noncoding RNAs upon UV irradiation.

## Results

### Cells elicit a multipronged DNA damage response upon UV-C irradiation

**Fig. 1:**
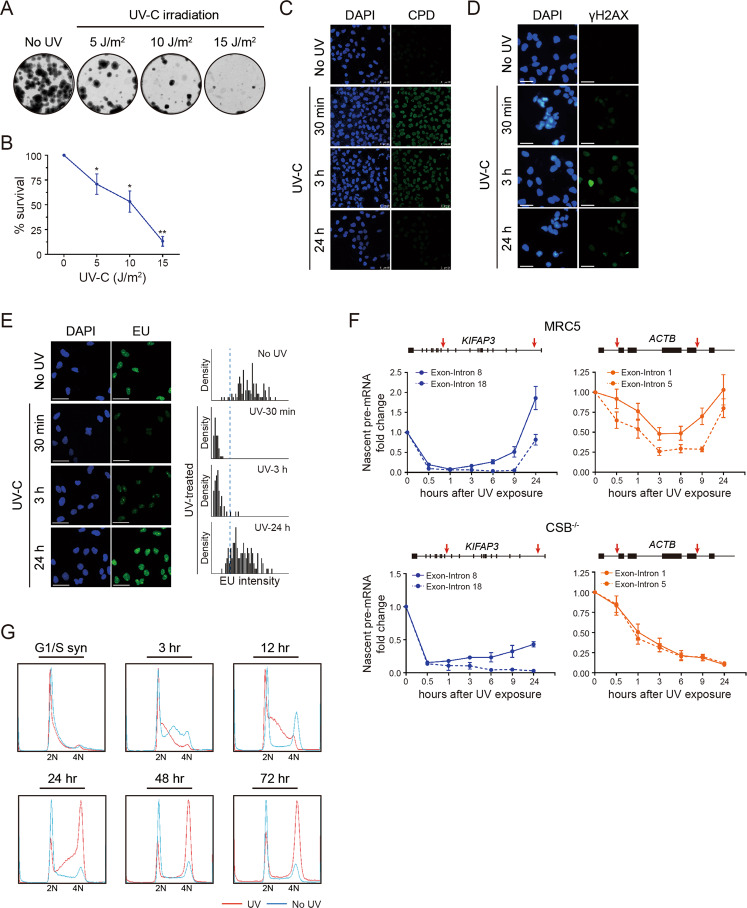
The ability of human fibroblasts MRC5_VA cells to repair UV-induced DNA lesions. **A** Representative clonogenic assay of human fibroblast-derived cell line MRC5_VA upon treatment of UV-C irradiation under the indicated dose. **B** Quantification of clonogenic assay shown in Fig. 1A. Data are means ± s.e.m. (*n* = 3 independent experiments) and are expressed in percentage of untreated control value. *P < 0.05, **P < 0.005; two-tailed unpaired Student’s *t* test. **C** Immunofluorescence of CPDs in human fibroblast cell line MRC5_VA after exposure to 10 J/m^2^ UV-C irradiation. Nuclear DNA was counterstained with DAPI. Scale bar = 50 μm. **D** Immunofluorescence of γH2AX in MRC5_VA cells after exposure to 10 J/m^2^ UV-C irradiation. Nuclear DNA was counterstained with DAPI. Scale bar = 50 μm. **E** Representative images of MRC5_VA cells at the indicated recovery time after 10 J/m^2^ UV-C irradiation. Nascent EU-labeled RNA shown in green and DAPI-stained nuclei in blue. Scale bar = 50 μm. Histogram plots of average EU incorporation after UV irradiation are shown on the right panel. Blue stippled lines demarcate thresholds of lowly transcribing cells. **F** Nascent pre-mRNA production in different regions of the human *KIFAP3* and *ACTB* genes in MRC5_VA and CSB^−/−^ cells after UV-C irradiation. Data are means ± s.e.m. (*n* = 3 independent experiments). **G** MRC5_VA cells were synchronized at G1/S phase prior to 10 J/m^2^ UV-C exposure, thymidine was withdrawn and cells were cultured in fresh medium. DNA profiles of MRC5_VA cells were analyzed by FACS at the indicated time after release from G1/S phase.

Human fibroblast-derived MRC5_VA cells exhibited a dose-dependent survival upon UV-C irradiation (Fig. 1A, B). Meanwhile, the result showed that MRC5_VA cells demonstrated largely complete repair of CPDs 24 h post irradiation (Fig. 1C). Furthermore, we assessed the kinetics of γH2AX, a sensitive marker for DNA double strand breaks, to monitor the replication and transcription stress in cells. In line with previous reports [14, 15], γH2AX was already seen as early as 30 min, continued to increase through 3 h and diminished 24 h post-irradiation (Figs. 1D, S1A and B). On the contrary, a high level of constitutive expression of γH2AX was observed in CS1AN cells (CSB-deficient cell line, so called CSB^−/−^) (Fig. S1A and B).

The staining of nascent transcripts with Ethynyl-Uridine (EU) indicated that the rapid transcriptional response was evident as early as 30 min upon UV-C irradiation, and transcription restored 24 h post-irradiation (Fig. 1E). This result was confirmed by RT-PCR analysis showing loss of newly-synthesized RNA transcripts by 30 min and a slow time-resolved arrival of RNAP II by 9–24 h post UV irradiation (Fig. 1F). Inversely, UV-induced arrested genes were unable to restore their initial mRNA expression level within 24 h post-UV treatment in CSB^−/−^ cells (Figs. 1F and S1C).

In the absence of exogenous stress, synchronized MRC5-VA cells resumed cycling immediately after release from G1/S phase, and 24 h later most of the cells re-entered a new cell cycle (profiles in blue in Fig. 1G). In contrast, the UV-treated human fibroblasts exhibited a significant S-phase arrest through the first 12 h after release (profiles in red in Figs. 1G and S1D), due to the activation of cell cycle checkpoint and transcription repression. Furthermore, inhibition of G2/M transition was also detected during the time course examined after DNA repair (profiles in red in Fig. 1G). This observation has been further confirmed by immunostaining of p-H3S10 protein. As shown in Fig. S1E, the level of p-H3S10 proteins was not significantly altered at 24 h after UV treatment, suggesting that the accumulation of cells at G2/M phase at 24 h post irradiation results from a G2/M checkpoint arrest instead of mitotic catastrophe. In CSB^−/−^ cells, on the other hand, UV exposure initiates apoptosis and results in death of the cells within 72 h (profiles in red in Fig. S1F).

Taken together, to portrait the UV-induced transcriptional responses, we examined three key events in our following analysis: the immediate response (30 min after UV exposure), early time point (3 h after UV irradiation) and the recovery phase (24 h post UV treatment).

### Cellular fractionation quantitatively captures transcriptional responses to UV-C irradiation

**Fig. 2:**
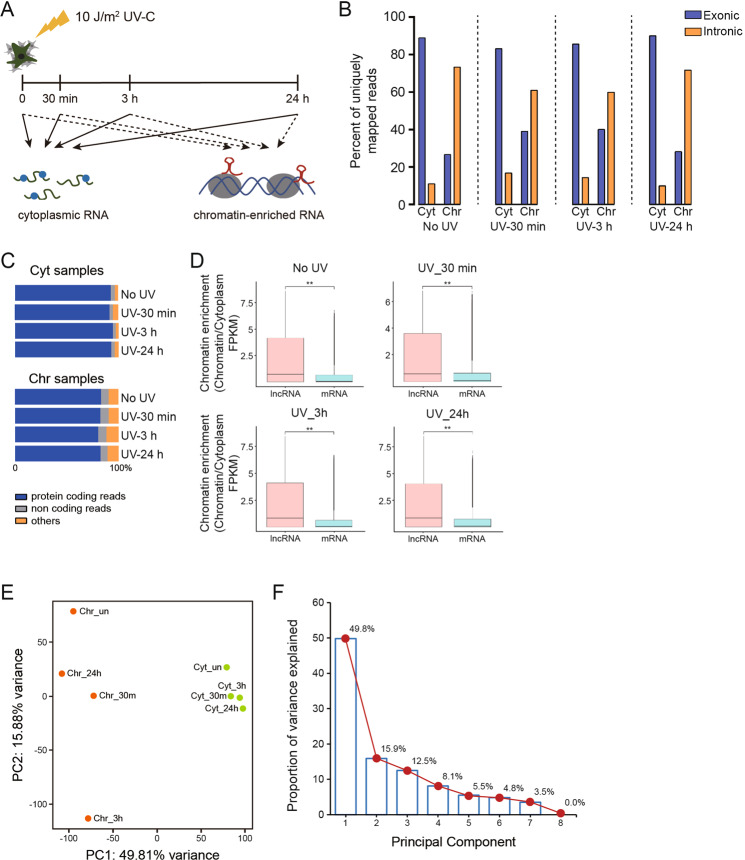
Chromatin-associated nascent transcripts in response to UV-C are enriched by cellular fractionation. **A** Schematic illustration of the cellular fractionation procedure. Cytoplasmic extract and chromatin pellet extract were isolated from human fibroblasts in triplicates, and then pools from the respective fractionation were sequenced. **B** The percentage of RNA sequencing reads mapping to intronic and exonic regions for all samples. Cyt, cytoplasm; Chr, chromatin. **C** Biotype distribution of all the transcripts identified by RNA-seq analysis generated from cytoplasm or chromatin fractions annotated relatively to human hg38. **D** Fold chromatin enrichment analysis of the indicated RNA classes (chromatin FPKM/cytoplasm FPKM) for the untreated and UV-irradiated human fibroblasts. Boxes span the lower to upper quartile boundaries, median is presented with a black line, *P* values are calculated by student’s *t* test, **P < 0.01. **E** Hierarchical clustering and PCA analysis of RNA-seq data from untreated and UV-irradiated MRC5_VA cells to separate all the samples into two groups. **F** The scree plot line indicates the amount of the total variance preserved by a principal component.

The cytoplasm and chromatin pellets were first extracted from MRC5_VA cells irradiated with 10 J/m^2^ UV-C and recovered by 30 min, 3 h, and 24 h (Fig. 2A). Then RNA-seq from the pool of three biological replicates was performed of the resulting cytoplasmic fraction and chromatin pellet extract, yielding at least 180 million uniquely mapped reads from each sample (Table S1). We first validated our fractionation by confirming robust chromatin enrichment of the two canonically chromatin-associated lncRNAs, *NEAT1* and *KCNQ1OT1* (Fig. S2A), and the cytoplasmic enrichment of the two mRNAs *ACTB* and *GAPDH* (Fig. S2B) under normal conditions. More importantly, our system can also detect the UV-dependent chromatin attachment of the DNA damage-induced transcription repressor *ATF3* (Fig. S2C) which has been documented to elicit transcription arrest following UV irradiation [10]. Notably, RNA samples from the chromatin pellet extracts showed high percentage of reads mapped to intronic regions as compared to the cytoplasmic extract (Fig. 2B). No major differences were seen in the read count distributions between untreated and UV-irradiated cells (Fig. 2C). LncRNAs as a whole are robust chromatin enriched relative to mRNAs (Fig. 2D). Principal component analysis of transformed RNA-seq count data separated chromatin-associated samples from the cytoplasmic extracts along the first principal component (Fig. 2E, F). Intriguingly, the variance in gene expression between the individual sample derived from the chromatin extract is substantially greater than the samples from the cytoplasmic fraction, suggesting a particular impact of UV irradiation on the chromatin-enriched transcriptional programs (Fig. 2E). Moreover, gene expression pattern of 24 h chromatin extract is similar to 3 h and non-irradiated samples (Fig. 2E), implying transcription restart during the late phase of DNA repair process. In CSB^−/−^ cells, on the contrary, 24 h chromatin extract is separated from other time points examined (Fig. S2D), suggesting a failure of transcription recovery due to the lacking of CSB protein.

### Transcription restore is predominantly observed in shorter genes after DNA repair

**Fig. 3:**
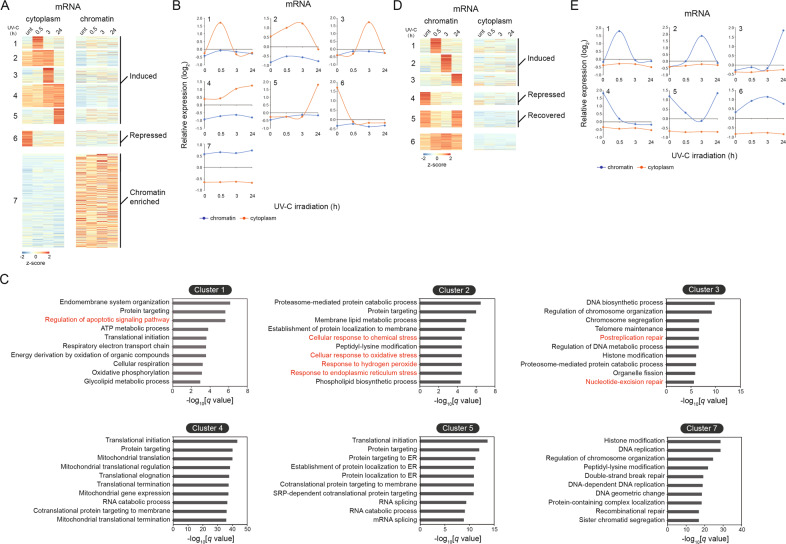
Dynamic transcriptional programs of mRNAs in response to UV-C irradiation. **A** Heat-map diagram showing the seven clusters of differentially expressed transcripts in the cytoplasm fraction following UV exposure. Color key represents relative expression on a log 2 scale. **B** The average relative transcript levels within each cluster presented in Fig. 3A are shown, with the log2 expression values on the y axis and time on the *x* axis. **C** Top 10 gene ontology enrichments of genes in clusters 2–5 and 7 with Bonferroni-corrected *P* values. **D** Heat-map diagram showing the six clusters of differentially expressed transcripts in the chromatin extract following UV exposure. Color key represents relative expression on a log 2 scale. **E** The average relative transcript levels within each cluster presented in Fig. 3D are shown, with the log2 expression values on the y axis and time on the x axis.

First of all, it is noteworthy that much more differentially expressed genes (DEGs) were observed in the chromatin extracts than in the cytoplasmic fractions in MRC5_VA cells recovered by 30 min after UV exposure, while in the cytoplasm extracts the number of DEGs reached a maximum at 24 h post irradiation (Fig. S3A and B). The fact that fewer genes in the cytoplasm responded during early time points is presumably due to the time required for pre-existing mRNAs to decay or processing of newly synthesized pre-mRNAs. Next, we divided 11,764 expressed RefSeq genes (with FPKM ≥ 1 in at least one fraction) into seven clusters based on their transcript expression profiles in the two subcellular fractions and four time points (Fig. 3A and Table S2). Inhibition of protein-coding gene expression during DDR was detected in cluster 6. Meanwhile, UV-induced genes were found in clusters 1–5 (Fig. 3A, B). UV exposure altered gene expression in the cytoplasm fraction within hours (Fig. 3A, B), and most induced genes in the early phase of DNA repair process were involved in DNA damage response (clusters 2 and 3 in Fig. 3C, Table S3), genes activated in the late response time, by contrast, encoded proteins that are enriched in the translational regulation process (clusters 4 and 5 in Fig. 3C, Table S3). Cluster 7 contains chromatin associated transcripts, with much lower expression levels in the cytoplasm relative to the other clusters. This cluster is dominated by genes required for histone modification and DNA replication (cluster 7 in Fig. 3C, Table S3). Notably, a group of genes encoding proteins that are involved in the regulation of apoptotic signaling pathway were enriched in cluster 1 (cluster 1 in Fig. 3C, Table S3). Among these genes, MAZ [16] and RACK1 [17] were previously proved to be important in the DNA damage response by modulating cell proliferation and apoptosis (Fig. S3C). The chromatin-enriched genes were further subdivided into six classes, on the basis of the temporal profiles (Fig. 3D, E and Table S4). GO term analysis revealed that none of the clusters was enriched for genes regulating DNA repair or cell cycle process (Table S5). Gene set enrichment analysis was also performed to functionally scrutinize the transcriptional changes upon UV irradiation in different cellular compartments. This analysis further confirms that the genes involved in DNA repair were enriched in response to UV treatment (Fig. S3D). Remarkably, the nucleotide excision repair genesets are enriched for genes in the cytoplasmic extracts after treatment, while the base excision repair genesets are enriched for chromatin-associated genes (Fig. S3D).

**Fig. 4:**
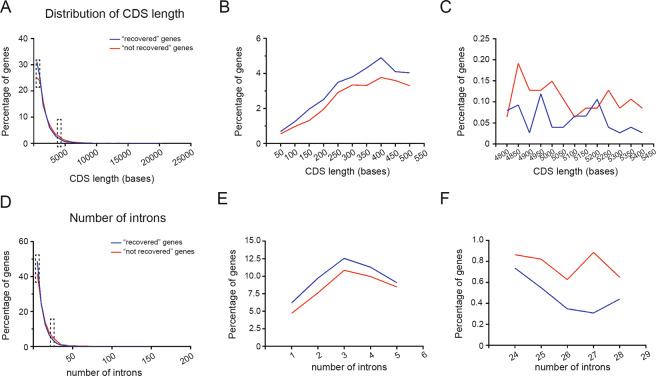
Features of the “recovered genes” upon UV exposure. **A** Distribution of CDS sizes for “recovered genes” and “non recovered genes”. Each data point represents the number of CDSs in each size class in increments of 100 bp. Where there are several CDS isoforms in a gene, they are all counted. **B** Enlarged view of the first 500 bases shown in Fig. 4A. **C** Enlarged view of the middle 600 bases shown in Fig. 4A. **D** Distribution of number of introns per gene. Each data point represents the number of genes having a specific number of introns. **E** Enlarged view of the genes with 1–5 introns shown in Fig. 4D. **F** Enlarged view of the genes with 24–28 introns shown in Fig. 4D.

In order to examine the transcription shutdown and recovery in accordance with DNA repair process, we grouped the expressed genes in the chromatin fraction into “recovered genes” (expression levels were reduced 30 min after UV irradiation and restored to normal levels 3 h or 24 h post-irradiation) and “not recovered genes” (expression levels were reduced 30 mins or 3 h after UV exposure and never restored at later time points). We noticed that there was a tendency for genes with transcription recovery in their coding regions to be short (Fig. 4A–C), with mean length of CDS being 1287 nt for “recovered genes”, compared to 1549 nt for “not recovered genes” (median length was 837 bp, compared to 1026 bp respectively). Moreover, “recovered genes” also preferred to have less introns than “not recovered genes” (Fig. 4D–F), with the mean number of introns being 8.5 for “recovered genes”, compared to 10.1 for “not recovered genes”. The biological meaning of enrichment for short genes being preferentially restored after UV exposure is definitely worth further investigation.

### Transcriptome profiling of lncRNAs in response to UV irradiation

**Fig. 5:**
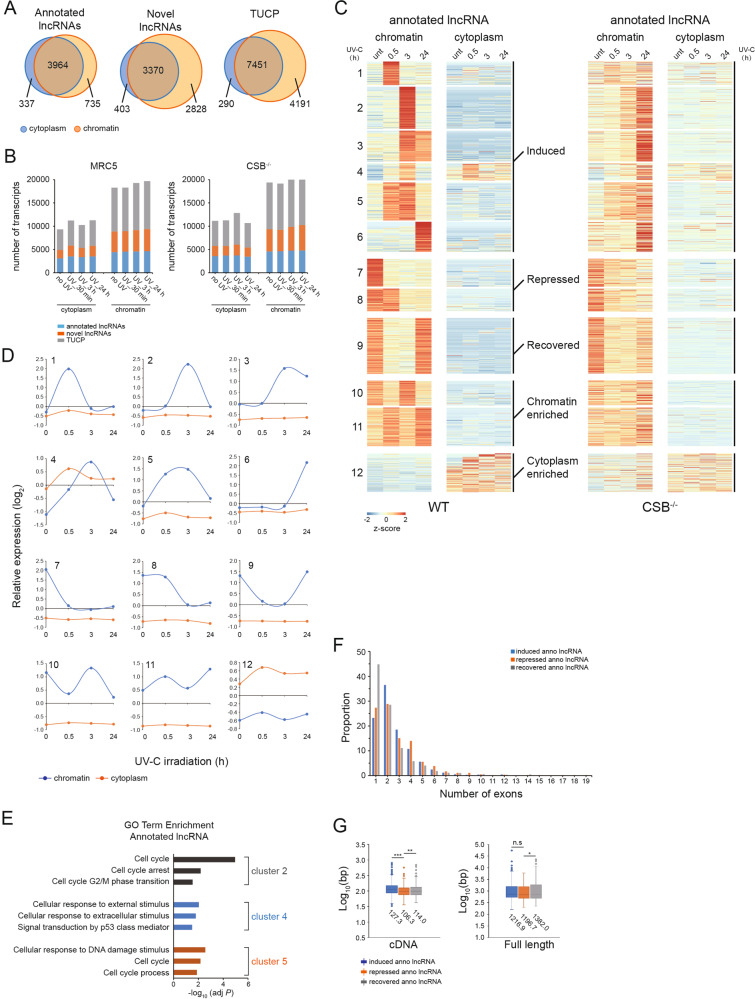
Spatial-temporal transcriptional dynamics of long noncoding RNAs upon UV exposure. **A** Distribution of identified annotated lncRNAs, novel lncRNAs and TUCPs in the cytoplasm and chromatin fractions. **B** Size of each biotype of non-coding RNA transcripts identified per cell line per condition is shown. **C** Heat-map diagram showing the twelve clusters of differentially expressed annotated lncRNA transcripts in the chromatin fraction following UV exposure. Color key represents relative expression on a log 2 scale. Data derived from the irradiated MRC5 cells shown on the left panel, data derived from the irradiated CSB^−/−^ cells shown on the right panel. **D** The average relative transcript levels within each cluster presented in Fig. 5B are shown, with the log2 expression values on the y axis and time on the x axis. **E** Top 3 gene ontology enrichments of the annotated lncRNAs in clusters 2, 4 and 5 with Bonferroni-corrected *P* values. **F** Distribution of number of exons for “induced”, “recovered” and “repressed” annotated lncRNAs. Each bar represents the number of noncoding transcripts having a specific number of exons. **G** Distribution of CDS and full sizes for the “induced”, “repressed” and “recovered” annotated lncRNA transcripts. *P* values are calculated by student’s *t*-Test, n.s *P* = 0.382, *P = 0.013, **P = 0.005, **P = 4.66e^−15^.

In order to reveal transcriptome landscape for lncRNAs during DNA damage repair process, we first applied cufflinks and scripture [18, 19] to identify novel lncRNAs (Fig. S4A). In total, 5036 annotated lncRNAs and 6,601 novel lncRNAs were obtained (Fig. 5A and Table S6). Surprisingly, we observed that the size of the TUCP (transcripts of uncertain coding potential, part of lncRNAs) is substantially greater than appreciated (Fig. 5A). Remarkably, the chromatin has the highest proportion of transcribed novel lncRNAs (~42.84% vs. ~14.59% for annotated lncRNAs), and TUCPs (~35.12%). Furthermore, the distribution of non-coding RNAs is not affected by UV-induced DNA damage (Fig. 5B). CSB deficiency did not have impact on the identification of non-coding RNAs upon UV (Fig. 5B). The relative abundance of these transcripts provides a valuable resource for further exploration of functional noncoding RNAs that are likely to operate at the chromatin interface (Table S6).

Compared to mRNAs, annotated lncRNAs also exhibited low mobility in that the UV-dependent changes in cellular distribution were only observed in 4.4% of annotated lncRNAs (cluster 4 in Fig. 5C, D). On the basis of the temporal profiles of their chromatin-associated transcripts, the UV-induced annotated lncRNAs were clustered into six groups (Fig. 5C, D, Table S7). The UV-induced novel lncRNAs and TUCPs were grouped into five classes (Fig. S4B–E, Tables S8 and S9). Interestingly, all these lncRNAs displayed undisturbed or mild changes in expression in the cytoplasm fraction. It has been well known that CSB regulates the transcriptional program of coding genes both under normal conditions and after UV irradiation [7, 20]. Surprisingly, in this study we found that, in CSB deficient cells, the chromatin-associated lncRNAs largely followed the same temporal profiles as in WT cells, but with a clearly detectable delay for the UV-induced lncRNAs (Figs. 5C, S4B and D).

We further explored the genes and pathways that the UV-induced annotated lncRNAs may regulate. Gene Ontology analysis indicated that the biological functions of mRNAs co-expressed with UV-induced annotated lncRNAs are associated with cell cycle regulation and cellular response to DNA damage (Fig. 5E showing the representative GO terms for lncRNAs in the clusters 2, 4, and 5 in Fig. 5C, Table S10). Notably, genes co-expressed with the UV-induced novel lncRNAs are enriched for functional groups in chromatin remodeling (Fig. S4F and Table S10). Finally, we analyzed the molecular features for the chromatin-enriched differentially expressed lncRNAs, including exon number, exon length, and transcript full length. Most of the “recovered” annotated lncRNAs showed an obvious tendency to have only one exon (44.8% for “recovered” annotated lncRNAs, compared with 27.3% of repressed annotated lncRNAs) (Fig. 5F). Interestingly, the average exon length of the “recovered” annotated lncRNAs is longer than that of the repressed lncRNAs (114.0 nt for the “recovered” annotated lncRNAs and 106.3 nt for the repressed lncRNAs) (Fig. 5G). Additionally, the average size of full-length transcripts for the “recovered” annotated lncRNAs is also longer than that of the repressed annotated lncRNAs (1382.0 nt for the “recovered” annotated lncRNAs and 1196.7 nt for the repressed lncRNAs) (Fig. 5G). Furthermore, the “recovered” novel lncRNAs enriched in the chromatin also tend to have longer length compared to the “not recovered” novel lncRNAs (Figs. S5A and B). These results imply that lncRNAs and mRNAs may have different mechanisms regulating their biogenesis, stability, and spatial-temporal expression patterns during DNA damage repair.

## Discussion

The experimental strategy described in this study is critical in its ability to simultaneously provide information about the action of transcripts (both mRNA and noncoding RNAs) that remain associated with chromatin and the changes the transcripts proceed to the cytoplasm. Besides effectively uncovering chromatin-associated relevant factors and pathways in the cellular response to UV-induced DNA damage, the study also provides an unprecedented overview of the diverse signatures of non-coding RNAs connected to the transcription-related DNA damage response. In particularly, we found that the majority of differentially expressed mRNA transcripts in the chromatin fraction are detected in the immediate response time (30 min) after UV exposure (Fig. S3A and B). Meanwhile, the inducible genes with cytoplasmic transcript levels increased at earlier time points are enriched for the biological processes including nucleotide-excision repair (Fig. 3C). We also observed that the majority of novel lncRNAs and TUCPs are chromatin enriched, while the annotated lncRNAs exhibit intermediate levels of chromatin attachment and cytoplasmic solubility (Fig. 5A, B). mRNAs with shorter length and less exon numbers are prefered to be recovered in response to DNA damage (Fig. 4), on the contrary, the “recovered” lncRNAs harbor longer length compared to the “not recovered” lncRNAs (Fig. 5F, G), implying distinct mechanistic possibilities that modulate transcriptional activity of different molecule species. Last but not least, the effect of CSB deficiency on the expression of noncoding RNAs after cellular stress is much milder than predicted (Figs. 5C, S4B and D).

One important question that must be carefully considered when interpreting our RNA-seq data is whether the abundant transcripts that attach to the chromatin are precursors to productive mature RNAs or whether instead they might be nonproductive “side-effect” transcripts because of the transcription shutdown induced by DNA damage. To address this question, absolute transcript abundances for the same gene in each subcellular compartment are of interest, which however cannot be determined due to the possible variability in RNA isolation efficiencies from the two fractions. Even though, the fact that the increase in chromatin transcripts is not followed by an increase in the cytoplasmic transcripts (Fig. 3D), argues against the productive precursors to mRNA. In fact, it has been proved that the synthesis of nascent RNAs ensures maximal transcription-driven repair throughout expressed genes [12]. Therefore, a detailed analysis of the molecular features and kinetics for these chromatin transcripts immediately increased upon UV can provide further evidence of unrestrained initiation of RNAP II through active genes after cellular stress.

It is broadly studied about the mechanisms and factors responsible for the dramatic shutdown of transcription genome wide after UV irradiation [6, 10, 21]. How the repression is inactivated to allow transcription to recover is still largely unknown. Here in this study, we identified “recovered” genes and “not recovered” genes within the time window we accessed using the DNA repair sufficient cells, and observed that shorter genes are preferred to be recovered first (Fig. 4). Regarding the occurrence of DNA lesions (500 T-T lesions per 10^6^ normal bases) [22], hypothetically, shorter genes contain less or no lesions than longer genes. Furthermore, an existing model suggests that transcription is spatially restricted for long periods, with the promoter-proximal 20–25 kb showing much more activity than the areas further downstream in longer genes after DNA damage, thereby virtually genes restored after DNA damage are short [13, 23, 24]. Conversely, noncoding RNAs associated with the chromatin that are recovered after UV irradiation are likely to be longer with undistinguished exon numbers compared to the “not recovered” noncoding RNAs (Figs. 5F, G, S5A and B). In fact, it has been suggested that mammalian pre-mRNAs and long noncoding RNAs employ thoroughly different transcription strategies [25]. More evidence is required to further evaluable whether the act of transcription on longer non-coding RNAs or the nature of these transcripts underlies their biological purpose. It is plausible that human fibroblasts selectively restrict a group of non-coding RNA turnover and so allow their adequate accumulation to facilitate DNA repair. Our results indicate that m^6^A RNA methylation actively participates in the modulation of cytoplasmic transcriptional activity of noncoding RNAs at 24 h in response to UV irradiation (Fig. S5E). The chromatin-enriched transcripts of noncoding RNAs are not associated with changes in m^6^A methylation. This result supports the idea that m^6^A modification of noncoding RNAs mainly regulates the post-transcriptional RNA metabolism including the transport, stability, and degradation of noncoding RNAs themselves.

In all, TC-NER is not merely a mechanism that would recruit the DNA repair factors to recognize and remove DNA lesions. Instead, a myriad of cellular activities is involved, among which transcription arrest and recovery has been elusive for several decades. Our results expand on earlier studies showing that induced chromatin transcripts after UV irradiation is not followed by delayed activation of genes in cytoplasm, providing new evidence to support the hypothesis that transcription activities may have an in cis function on TC-NER. Therefore, a future challenge is to uncover the precise schedule of all events required to repair DNA lesions. How is transcriptional program linked to cell-cycle arrest and TC-NER? What are the key regulatory mechanisms of global transcription shut down and favored transcription restart of shorter mRNAs? What are the emerging roles of noncoding RNAs in regulating the balance between continuous loads of fresh RNAP II and degradation of arrested RNAP II? New tools to map sites of DNA damage and its repair for the entire genome at single-nucleotide resolution have been developed within the recent five years [26, 27]. DNA repair-related XR-seq combined with RNA-seq from chromatin and cytoplasmic fractions can help decipher how transcriptional control contributes to DNA repair and vice versa.

## Materials and methods

### Cell culture and treatment conditions

SV40-transformed normal human fibroblast MRC5 cells and CS1AN (CSB deficient cell line derived from CS patient) fibroblasts [20] were grown in Dulbecco’s modified Eagle’s medium (Gibco, 12430104) containing 10% fetal calf serum (Gibco, 10100147C) and antibiotics in 5% CO_2_ at 37 °C. Cells were seeded in dishes and grown to confluence. Before UV irradiation, cells were washed with PBS and irradiated with UV-C light (254 nm) at a required dosage, ranging from 5 to 15 J/m^2^. After irradiation, cells were grown in fresh medium for various time periods.

### Clonogenic survival assay after UV-C treatment

Exponentially growing human fibroblast-derived MRC5_VA cells were plated in 6-well tissue culture plates (1 × 10^4^ cells seeded per well). After incubation for 24 h, the cells were exposed to UV-C irradiation from 0 to 15 J/m^2^, and the cultures were maintained until surviving cells formed colonies. Cells were fixed and stained with a mixture of 0.5% crystal violet in absolute methanol for 15 min. Colonies were scored, and the surviving fractions for each dose were calculated.

### Immunofluorescence

For UV treatment, MRC5_VA cells were exposed to 10 J/m^2^ UV-C irradiation and recovered by 30 min, 3 h and 24 h before subjected to immunofluorescence. After fixation with 4% (vol/vol) paraformaldehyde in PBS for 15 min at room temperature, cells were permeabilized in 1× PBS containing 0.1% Triton X-100 and blocked with blocking solution [1× PBS containing 0.01% Triton X-100, 10% (vol/vol) FBS, and 3% (wt/vol) BSA] for 1 h. Antibodies against CPD (1:500 dilution, Cosmobio, TDM-2), γH2AX (1:1000 dilution, Santa Cruz, sc-517348), or p-H3S10 (1:1000 dilution, CST, D7N8E) in blocking solution were then added and incubated for 1 h at room temperature, followed by washing and incubation with fluorophore-conjugated corresponding secondary antibodies (Alexa 488 anti-rabbit, 1:1000 dilution, Life Technologies, A32723). Coverslips were counterstained and mounted on slides using mounting medium with DAPI (Vector Laboratories, Inc. Peterborough, UK). Images were acquired on a laser scanning confocal microscope (Carl Zeiss, LSM700).

### 5′ Ethynyl Uridine staining

EU staining to detect newly synthesized RNA was performed according to the manufacturer’s instructions (Click-iT RNA imaging Kits, Invitrogen, C10329). Briefly, cells were exposed to 10 J/m^2^ UV-C irradiation and incubated for the indicated time. Media was replaced with fresh media containing 0.75 mM 5′ EU and cells were incubated for 2 h. EU-containing media was then removed and cells were fixed in PBS buffered formaldehyde (3.7%) for 45 min at room temperature, washed once with PBS and followed by permeabilization with 0.5% Triton X-100 diluted in PBS for 30 min. Cells were washed once with PBS then Alexa Fluor 488 Azide fluorophores were covalently attached to EU-containing RNA by click reaction for 1 h at room temperature. Cells were then counterstained and mounted with mounting medium containing DAPI. Automated image acquisition of 5 fields per slide was performed (Carl Zeiss, LSM700).

### qRT-PCR of nascent pre-mRNA synthesis

Total RNA was extracted from cultured cells with an RNeasy Mini Kit (Qiagen, 74104), according to the manufacturer’s instructions. The integrity of the RNA was tested on a denaturing agarose gel. RNA quality and quantity were also assessed with a Nanodrop spectrophotometer (Thermo Fisher Scientific). For quantitative RT-PCR analysis, single-stranded cDNA was synthesized from 200 ng of total RNA using a TaqMan Reverse Transcription Kit (Invitrogen, N8080234). Primers used to amplify exon-intron junctions for the human *KIFAP3* and *ACTB* gene were described as follows:

Human *KIFAP3* Ex-In8_For: ACAGGAACAGCTATTACGAGGT

Human *KIFAP3* Ex-In8_Rev: CCCATGCTAAAGACAGACGAAC

Human *KIFAP3* Ex-In18_For: CCCTGCTAGGAAGAGAATCTTGGT

Human *KIFAP3* Ex-In18_Rev: TGGTTGGCCAAAGCCATCCATT

Human *ACTB* Ex-In1_For: CCGACCAGTGTTTGCCTTTT

Human *ACTB* Ex-In1_Rev: GCGGCGATATCATCATCCAT

Human *ACTB* Ex-In5_For: GTGTCACATCCAGGGTCCTC

Human *ACTB* Ex-In5_Rev: TCGTCATACTCCTGCTTGCT

### Cell cycle analysis by FACS

Exponentially multiplying cells were incubated for 12 h in maintenance media supplemented with 2 mM thymidine. Cells were then washed three times with PBS and incubated for 10 h in maintenance media, followed by additional 12 h incubation in maintenance media supplemented with 2 mM thymidine. A total of 1–5 × 10^5^ cells were collected by centrifugation at 300 × *g* for 3 min and then washed and resuspended in 100 μl of 1× PBS containing 1% FBS. Cold methanol (1 mL) was added to the cell suspension drop-wise while vortexing the open tube at minimum speed on a Vortex-Genie 2 vortexer (Scientific industries, Inc.). Cells were fixed at 4 °C from 1 h up to 1 week. After this treatment, cells were washed once in 1× PBS + 1% FBS and resuspended in 0.5–1 mL of staining solution (1× PBS, 1% FBS, 25 μg/mL propidium iodide, and 10 μg/mL RNase A). Samples were incubated at 37 °C for 30 min in the dark. Before acquisition on the flow cytometer cell scanner, cells were filtered through a 0.45 μm mesh.

### Cellular fractionation

Cellular fractionation was performed as described before [28]. Briefly, human fibroblast cell line MRC5_VA cells grown to 80% confluence were scraped and collected by centrifugation (200 × *g*, 5 min, 4 °C). The cell pellets were resuspended in cell lysis buffer (10 mM Tris-HCl pH 7.4, 150 mM NaCl, 0.15% NP-40) and incubated on ice for 5 min. The cell lysate was overlayed on top of 2.5 volume of the sucrose buffer (10 mM Tris-HCl pH 7.4, 150 mM NaCl, 24% sucrose) by slowly pipetting into the wall of the tube. The mixture was centrifuged (3500 × *g*, 10 min, 4 °C) and the supernatant was collected as the cytoplasmic fraction. The nuclei pellets were rinsed with ice-cold PBS-EDTA and resuspended sequentially in glycerol buffer (20 mM Tris-HCl pH 7.4, 75 mM NaCl, 0.5 mM EDTA, 50% Glycerol) and the same volume of Urea buffer (10 mM Tris-HCl pH 7.4, 1 M Urea, 0.3 M NaCl, 7.5 mM MgCl_2_, 0.2 mM EDTA, 1% NP-40), the sample was mixed by vortexing for 4 s and incubated on ice for 2 min. The chromatin-RNA complex was precipitated by centrifugation (13,000 × *g*, 2 min, 4 °C) and resuspended in TRIzol reagent. The pellet was solubilized by passing through a 21-gauge needle. For RNA isolation from different fractions, TRIzol reagent was added directly to the cytoplasmic extract and RNA was isolated according the manufacturer’s instruction.

### RNA-seq library construction and sequencing

All RNA samples had a RIN (RNA Integrity Number) value of greater than 9.8. cDNA libraries were generated by using the TruSeq Stranded Total RNA Sample Prep kit (Illumina, RS-122-2001) with Ribo-zero gold (Epicentre, MRZG12324). Libraries were quantified fluorometrically using Qubit^®^ dsDNA HS Assay Kits on a Qubit 2.0 Fluorometer (Invitrogen, Q32854). All libraries (>2 nM/μl) were sequenced 150 bp paired-end on Illumina HiSeq XTen according to the manufacturer’s instruction to a depth of 210–410 million reads.

### Processing, analysis, and graphic display of RNA-seq data

Raw reads were pre-processed with sequence grooming tool FASTQC followed by sequence alignment using HISAT2 [29]. Transcript levels were quantified as fragments per kilobase of transcript per million mapped reads (FPKM) generated by TopHat/Cufflinks [30]. SAMtools [31] were used to convert sam files to bam files in order to make raw data visible on IGV (Integrative Genomics Viewer) or UCSC Genome Browser. Transcript levels were converted to the log-space by taking the logarithm to the base 2. R studio was used to run custom R scripts to perform box plots, principal component analysis, scatter plots, dendrograms, and heatmaps. Generally, ggplot2 and gplots packages were used to generate these data graphs. Samples were analyzed through DESeq2 [32] to obtain log2 fold change and its respective p value. Differentially expressed transcripts have been identified on these transformed values by using the criteria of log2 (Fold Change) ≥ 1 and padj < 0.05. Gene ontology analysis was performed using clusterProfiler [33].

### Identification of novel lncRNAs and TUCP

The known noncoding RNAs expressed in at least one fraction were identified by blasting the transcripts against the NONCODE v6.0 database [34] using the following selection criteria: identity >0.9, coverage >0.8, and *E* value < 10^5^. These transcripts were named as the ID number in the NONCODE v3.0 databse.

Based on the features of lncRNA and the functional characteristics of noncoding proteins, a series of stringent screening conditions were established to identify novel lncRNAs and TUCPs: (1) Cuffmerge was used to merge the transcripts which obtained by splicing and remove transcripts with uncertain directions and <200 bp in length; (2) Cuffcompare was used to filter out transcripts that overlap with the database annotation exon region; (3) Cuffquant was applied to calculate the expression level of each transcript and transcripts with FPKM ≥ 0.5 were selected; (4) Four analysis tools, including CNCI (Coding-Non-Coding-Index, v2), CPC (encoding potential calculator, 0.9-r2), Pfam Scan (v1.3) and phyloCSF were used to predict the coding potential of the transcripts.

## Supplementary information

Supplementary File

Figure S1

Figure S2

Figure S3

Figure S4

Figure S5

Dataset 1

Dataset 2

Dataset 3

Dataset 4

Dataset 5

Dataset 6

Dataset 7

Dataset 8

Dataset 9

Dataset 10

aj-checklist

## Data Availability

RNA-seq data used in this study are available under GEO: GSE184408. ATAC-seq and meRIP-seq data used for the integrated analysis in this study have been published [35, 36] and are available under GEO: GSE161793.
