# Supplementary material for: Cellular fractionation reveals transcriptome responses of human fibroblasts to UV-C irradiation
Source: Cell Death Dis. 2022 Feb 24;13(2):177. doi: 10.1038/s41419-022-04634-x (PMC8873393; doi:10.1038/s41419-022-04634-x)
Supplement: Supplementary file 1 — Supplementary File [file 41419_2022_4634_MOESM1_ESM.docx]

**Supplementary Figure Legends**

**Figure S1. DNA repair deficiency in CSB-deficient cells after UV irradiation, Related to Figure 1**

**(A)** Immunostaining of γ-H2AX (green) and DNA (using DAPI; blue) in MRC5 and CSB^-/-^ cells in response to 10 J/m^2^ UV-C irradiation. **(B)** Quantification of γ-H2AX foci detected in (A). A minimum of 50 cells per cell line per condition were analyzed. Points represent ± SEM from three independent experiments. **(C)** Representative images of CSB^-/-^ cells at the indicated recovery time after 10 J/m^2^ UV-C irradiation. Nascent EU-labeled RNA shown in green and DAPI-stained nuclei in blue. Scale bar = 50 μm. **(D)** Summary of the % of cells in each phase of the cell cycle for MRC5_VA cells 12 h released from the G1/S phase with or without UV irradiation. Significant differences (* *p* < 0.05, ** *p* < 0.001) are indicated. Bars are mean ± SEM from three independent experiments. **(E)** MRC5_VA cells were treated with or without UV (10 J/m^2^) upon the time released from G1/S synchronization. At the indicated time, the cells were incubated with anti-p-H3S10. Mitotic index (the percentage of mitotic cell number/total cell number) was counted under a fluorescence microscope. Results were obtained from three independent experiments and the bar represents the mean ± SEM. *p* value indicates no significant difference between untreated and UV treated samples or 3 h and 24 h post UV treatment. **(F)** CSB^-/-^ cells were synchronized at G1/S phase prior to 10 J/m^2^ UV-C exposure, thymidine was withdrawn and cells were cultured in fresh medium. DNA profiles of CSB^-/-^ cells were analyzed by FACS at the indicated time after release from G1/S phase.

**Figure S2. Chromatin-associated RNAs are efficiently separated from cytoplasmic RNAs by cellular fractionation, Related to Figure 2**

**(A-C)** Cytoplasmic extract and chromatin pellet extract from MRC5_VA cells aligned to the genome (hg38) and presented as coverage (normalized reads per million) as a function of chromosomal coordinate at the indicated lncRNA or protein-coding gene loci. Reads mapped to the sense strands are depicted above the axis. The integrated ratios are presented in the bar charts to the right for each RNA. Cyt, cytoplasm; Chr, chromatin. **(D)** Hierarchical clustering and PCA analysis of mRNAs detected from RNA-seq data for non-irradiated and UV-irradiated MRC5_VA and CSB^-/-^ cells. Chr, chromatin; Cyt, cytoplasm.

**Figure S3. Differentially expressed mRNAs in UV-irradiated cells, Related to Figure 3**

**(A)** Scatter plot representing the LogFC vs. Adj *p*-value of mRNAs differentially expressed upon UV treatment at the three indicated time points respectively. Dots are color coded based on expression profile of each gene. Blue represents mRNA transcripts without significant changes after UV irradiation, red represent upregulated genes and green represent downregulated genes. **(B)** Summery of the number of up- and down-regulated genes in the UV-irradiated cells versus untreated cells. Fold change ≥ 1.5 and adjusted *p* value < 0.05 were used as the threshold to judge the significance of gene expression difference. **(C)** Verification of RNA-seq expression of selected genes involved in the regulation of cellular apoptosis via qRT-PCR. **(D)** Heatmap showing the GSEA for DNA repair gene sets enriched in cytoplasm versus chromatin extracts in response to UV-C irradiation.

**Figure S4. Transcriptome profiling of novel lncRNAs and TUCP in response to DNA damage, Related to Figure 5**

**(A)** Features of identified novel lncRNAs and TUCP. Cumulative frequency plot of PhastCons conservation scores for mRNA, annotated lncRNA and novel lncRNA genes. Length, exon number and ORF distributions for mRNA, annotated lncRNA and novel lncRNA genes are shown on the right panel. **(B)** Heat-map diagram showing the twelve clusters of differentially expressed novel lncRNA transcripts in the chromatin fraction following UV exposure. Color key represents relative expression on a log 2 scale. Data derived from the irradiated MRC5 cells shown on the left panel, data derived from the irradiated CSB^-/-^ cells shown on the right panel. **(C)** The average relative transcript levels within each cluster presented in Figure S5B are shown, with the log2 expression values on the y axis and time on the x axis. **(D)** Heat-map diagram showing the eight clusters of differentially expressed TUCP transcripts in the chromatin fraction following UV exposure. Color key represents relative expression on a log 2 scale. Data derived from the irradiated MRC5 cells shown on the left panel, data derived from the irradiated CSB^-/-^ cells shown on the right panel. **(E)** The average relative transcript levels within each cluster presented in Figure S5D are shown, with the log2 expression values on the y axis and time on the x axis. **(F)** Top 3 gene ontology enrichments of the novel lncRNAs in cluster 4 with Bonferroni-corrected *P* values.

**Figure S5. Association analysis between gene expression and epigenetic regulation during DDR, Related to Figure 5**

**(A)** Exon number distribution for “induced”, “repressed” and “recovered” novel lncRNAs. Each bar represents the number of noncoding transcripts having a specific number of exons. **(B)** Distribution of CDS and full sizes for “induced”, “repressed” and “recovered” novel lncRNA transcripts. **(C-D)** The correlation of chromatin accessibility and RNA-seq measured gene expression for protein-coding genes (C) and noncoding RNA transcripts (D). **(E-F)** The correlation of m^6^A methylation and RNA-seq measure gene expression for noncoding RNAs in the cytoplasm (E) and chromatin fractions (F).

**Supplementary Table**

**Table S1**. RNA-seq mapping statistics

**Table S2**. Normalized FPKM values for transcripts in each cluster, Related to Figure 3A

**Table S3**. List of significant GO terms for each subcluster, Related to Figure 3C

**Table S4**. Raw FPKM values for transcripts in each cluster, Related to Figure S3C

**Table S5**. List of significant GO terms for each subcluster, Related to Figure 3D

**Table S6**. FPKM values for all noncoding RNA transcripts identified in MRC5_VA cells in response to DNA damage, Related to Figure 5A

**Table S7**. Normalized FPKM values for annotated lncRNA transcripts identified in MRC5_VA cells in each cluster, Related to Figure 5B

**Table S8**. Normalized FPKM values for novel lncRNA transcripts identified in MRC5_VA cells in each cluster, Related to Figure S5B

**Table S9**. Normalized FPKM values for TUCP transcripts identified in MRC5_VA cells in each cluster, Related to Figure S5D

**Table S10**. List of significant GO terms for each subcluster, Related to Figure 5D and Figure S4F
