# Supplementary figures and images for: Cellular fractionation reveals transcriptome responses of human fibroblasts to UV-C irradiation
Source: Cell Death Dis. 2022 Feb 24;13(2):177. doi: 10.1038/s41419-022-04634-x (PMC8873393; doi:10.1038/s41419-022-04634-x)

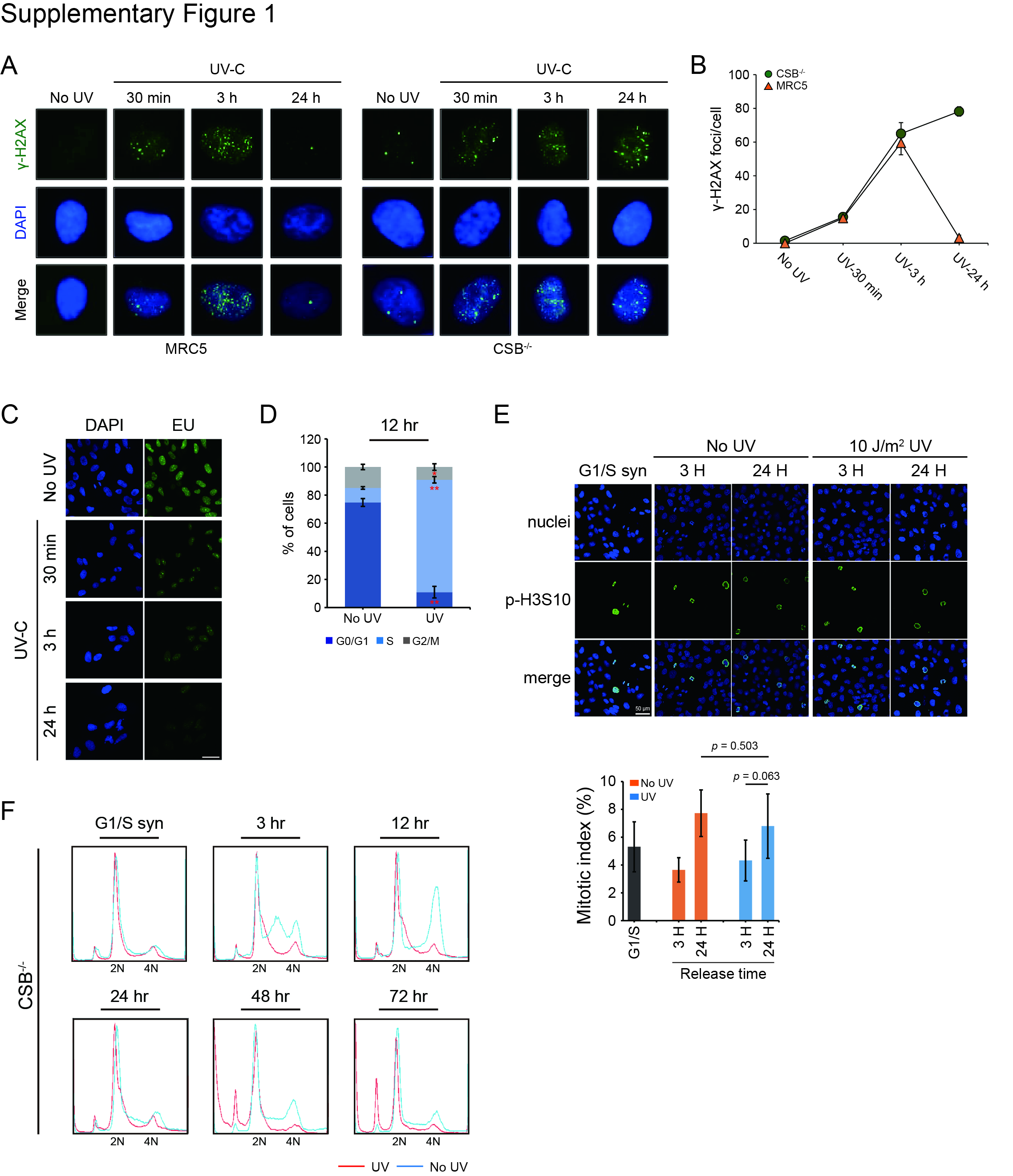

Supplement: Supplementary file 2 — Figure S1 [file 41419_2022_4634_MOESM2_ESM.jpg]

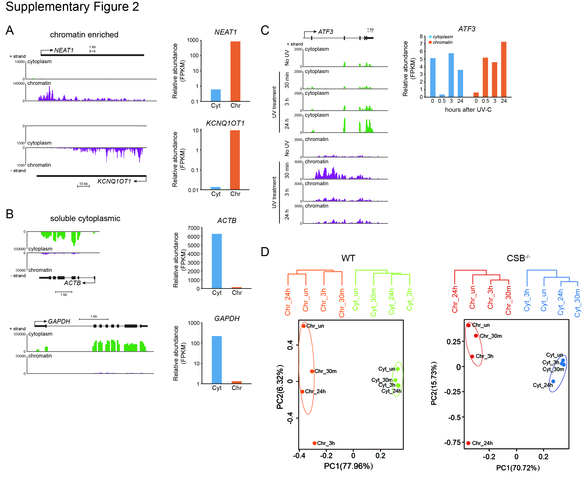

Supplement: Supplementary file 3 — Figure S2 [file 41419_2022_4634_MOESM3_ESM.jpg]

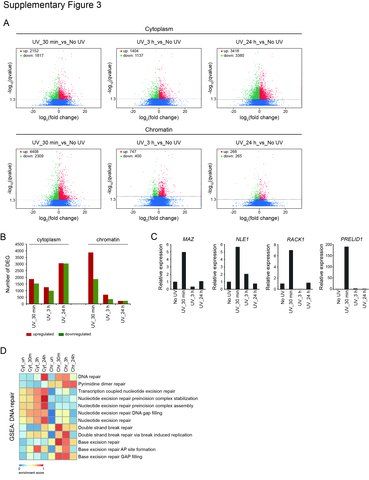

Supplement: Supplementary file 4 — Figure S3 [file 41419_2022_4634_MOESM4_ESM.jpg]

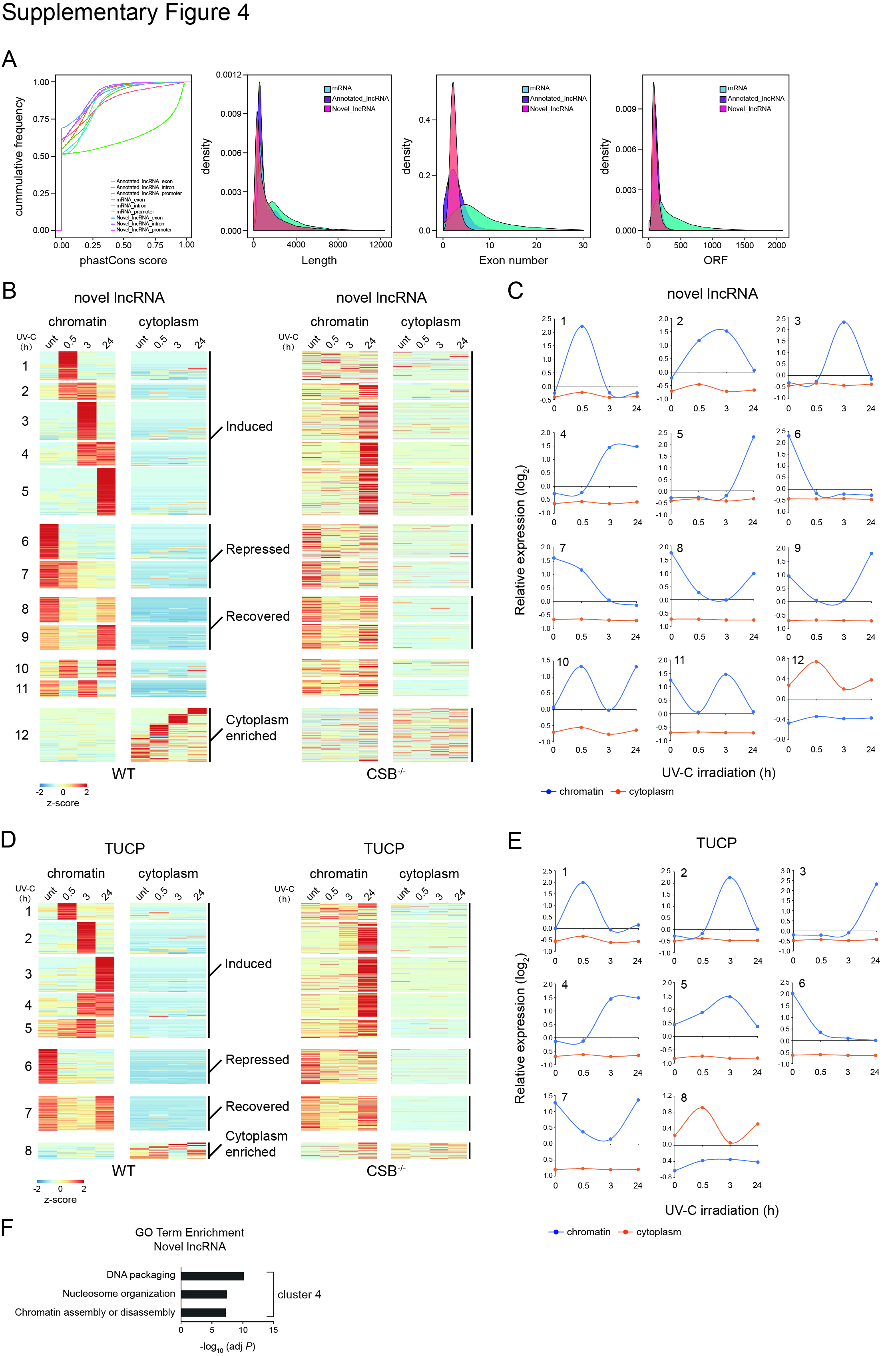

Supplement: Supplementary file 5 — Figure S4 [file 41419_2022_4634_MOESM5_ESM.jpg]

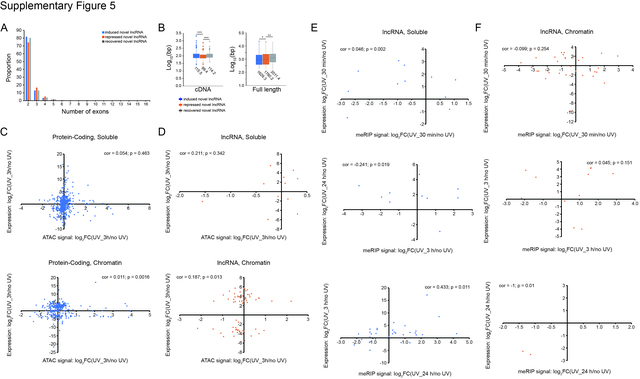

Supplement: Supplementary file 6 — Figure S5 [file 41419_2022_4634_MOESM6_ESM.jpg]
